# Evaluation of Maxillary Sinus Septa Using Cone Beam Computed Tomography (CBCT): A Retrospective Study

**DOI:** 10.7759/cureus.68157

**Published:** 2024-08-29

**Authors:** Rajat Verma, Nisha Dua, Rajesh Gupta, Mayank Jain, Mridula, Monika Gupta

**Affiliations:** 1 Oral Medicine and Radiology, Swami Devi Dyal Hospital and Dental College, Panchkula, IND; 2 Oral and Maxillofacial Radiology, Swami Devi Dyal Hospital and Dental College, Panchkula, IND

**Keywords:** dental implants, perforation risk, schneiderian membrane, cone beam computed tomography (cbct), septa, maxillary sinus

## Abstract

Background: Maxillary sinus septa, which are bony structures dividing the sinus cavity, can pose challenges during sinus lift or implant surgeries by potentially causing perforation of the Schneiderian membrane. This study aimed to estimate the prevalence, height, location, orientation, and risk of perforation of the maxillary sinus septa using cone beam computed tomography (CBCT).

Materials and methods: This retrospective, cross-sectional study utilized CBCT (NewTom CBCT machine, of which the scan parameters were 90 KvP, 8 mAs, and 14 s exposure with a field of view (FOV) of 8×8 cm and a 0.2 mm^3^ voxel size) images of 300 maxillary sinuses from patients aged >18 years, obtained from Swami Devi Dyal Hospital and Dental College in Panchkula, India. Scans were analyzed for the presence, height, location, orientation, and risk of septal perforation. The data were categorized based on age, sex, and dentition status. Statistical analyses were performed to assess the prevalence, configuration, and risk factors.

Results: The prevalence of maxillary sinus septa was 21.33%, with the majority showing a single septum (90.63%). Septa were predominantly found in the middle region (48.44%), with bucco-palatal orientation (93.75%) being more common than anteroposterior. The mean septa height was 6.16 mm. The perforation was classified as moderate (48.4%), low (46.8%), or high (4.6%). Class III septa were associated with the highest risk of perforation.

Conclusion: This study highlights a significant prevalence of maxillary sinus septa with variations in height, orientation, and location. The risk of perforation varies with the septa configuration and orientation. CBCT is essential for identifying these anatomical features to minimize surgical complications and to guide preoperative planning.

## Introduction

The maxillary sinus, one of the largest paranasal sinuses, is crucial in dental and maxillofacial surgeries because of its proximity to the upper jaw. Its anatomy can significantly impact surgical procedures, particularly those involving the placement of dental implants or sinus lifts. One of the key anatomical features relevant to these procedures is the presence of the bony septa within the sinus cavity. Septa are osseous structures that divide the sinus into smaller compartments, which can pose challenges during surgical interventions [1].

The prevalence of maxillary sinus septa varies across different populations. According to Al-Zahrani et al., the incidence of septa ranges from 9.5% to 69% in different studies, highlighting the variability in findings across geographic and demographic groups [2]. Septa can affect surgical outcomes, particularly in procedures such as sinus lifts, where their presence might complicate the elevation of the sinus membrane and increase the risk of complications such as Schneiderian membrane perforation [3]. Schneiderian membrane perforation is a common complication during maxillary sinus surgery, which can lead to increased postoperative morbidity, delayed healing, and the risk of sinus infections [4].

Cone beam computed tomography (CBCT) has become the gold standard for evaluating sinus anatomy because of its high resolution and ability to provide detailed three-dimensional images of the maxillary sinus [5]. Unlike traditional panoramic radiography, CBCT can effectively visualize the presence and configuration of septa, allowing for more accurate preoperative planning. The ability to classify septa based on their height, orientation, and location is crucial for predicting the risk of complications and tailoring surgical approaches [3].

Previous studies have documented various septa configurations, including single, multiple, and complex forms. Assari et al. reported that the majority of sinus septa are single, but complex septa can present significant challenges during surgical procedures [6]. The height and orientation of septa also play a role in their clinical significance, with bucco-palatal orientations being more common and potentially affecting the ease of surgical access [2]. Understanding these anatomical variations and their implications is essential for improving surgical outcomes and minimizing risks.

This study aimed to evaluate the prevalence, height, location, and orientation of the maxillary sinus septa using CBCT. Additionally, it sought to assess the risk of Schneiderian membrane perforation based on a modified classification system. By analyzing these factors, this study aimed to provide valuable insights for preoperative planning and risk management in maxillary sinus surgeries.

## Materials and methods

This retrospective cross-sectional study was approved by the Institutional Ethical Committee of Swami Devi Dyal Hospital and Dental College in Panchkula, India (approval number: SDDHDC/IEC/Mar2022/004). CBCT images of 300 maxillary sinuses were obtained retrospectively from the archives of a radiology center in Chandigarh, covering the study period from May 2022 to May 2023. The study included subjects over 18 years of age, and all scans were conducted on a NewTom CBCT machine with parameters set at 90 kVp, 8 mAs, and a 14-second exposure time. The field of view (FOV) was 8×8 cm with a voxel size of 0.2 mm³, and the scans were captured in high-definition mode with metal artifact reduction software. Data were reconstructed in 1 mm interval slices. Demographic data, including age and sex, were retrieved from information registered in the CBCT software before the procedure. The dental status of the participants was categorized as dentate (complete set of maxillary posterior teeth), partially dentate (at least one premolar and one molar present), or edentate (absence of all posterior teeth) in the maxillary region.

The study's inclusion criteria focused on images of patients aged >18 years without significant changes in the maxillary sinus. In contrast, the exclusion criteria were images with artifacts or those showing significant pathology, such as benign or malignant neoplasms and bone dysplasia, affecting the sinus or maxillary region. The final sample size was determined to be 300 after appropriate calculations.

Methodology

Image analysis was done to evaluate the prevalence, height, location, and orientation of the septa of the maxillary sinus and its correlation with age, gender, and dentition status. All the parameters were viewed and measured by two examiners and recorded on a predesigned proforma.

Prevalence of septa

The prevalence of septa was evaluated using axial, coronal, and sagittal CBCT sections.

Height of septa

**Figure 1:**
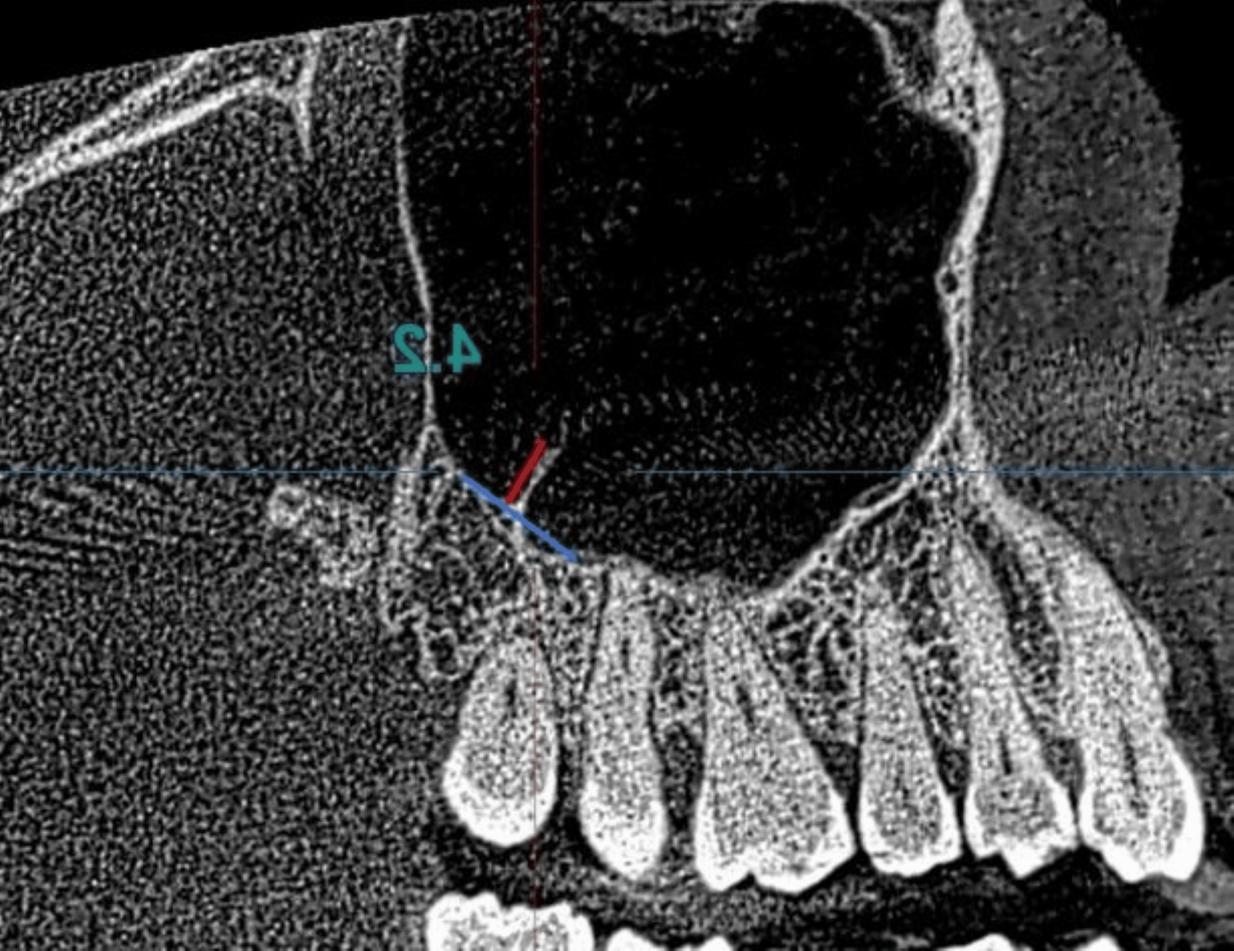
The measurement of the height of the septa

The height of the septa (Figure 1) was measured in the sagittal view, a tangent was drawn to the floor of the maxillary sinus at the base of the septa, and the height was measured by a perpendicular line drawn from the top of the septa to the tangent using the measurement tool of the software.

Location of septa

The positions of the maxillary septa were documented following the guidelines established by Kim et al. (2006) [7] with delineations for the anterior (mesial to the second premolar), middle (distal to the second premolar to the second molar), and posterior (distal to the second molar). A septum is observed in the middle region.

**Figure 2:**
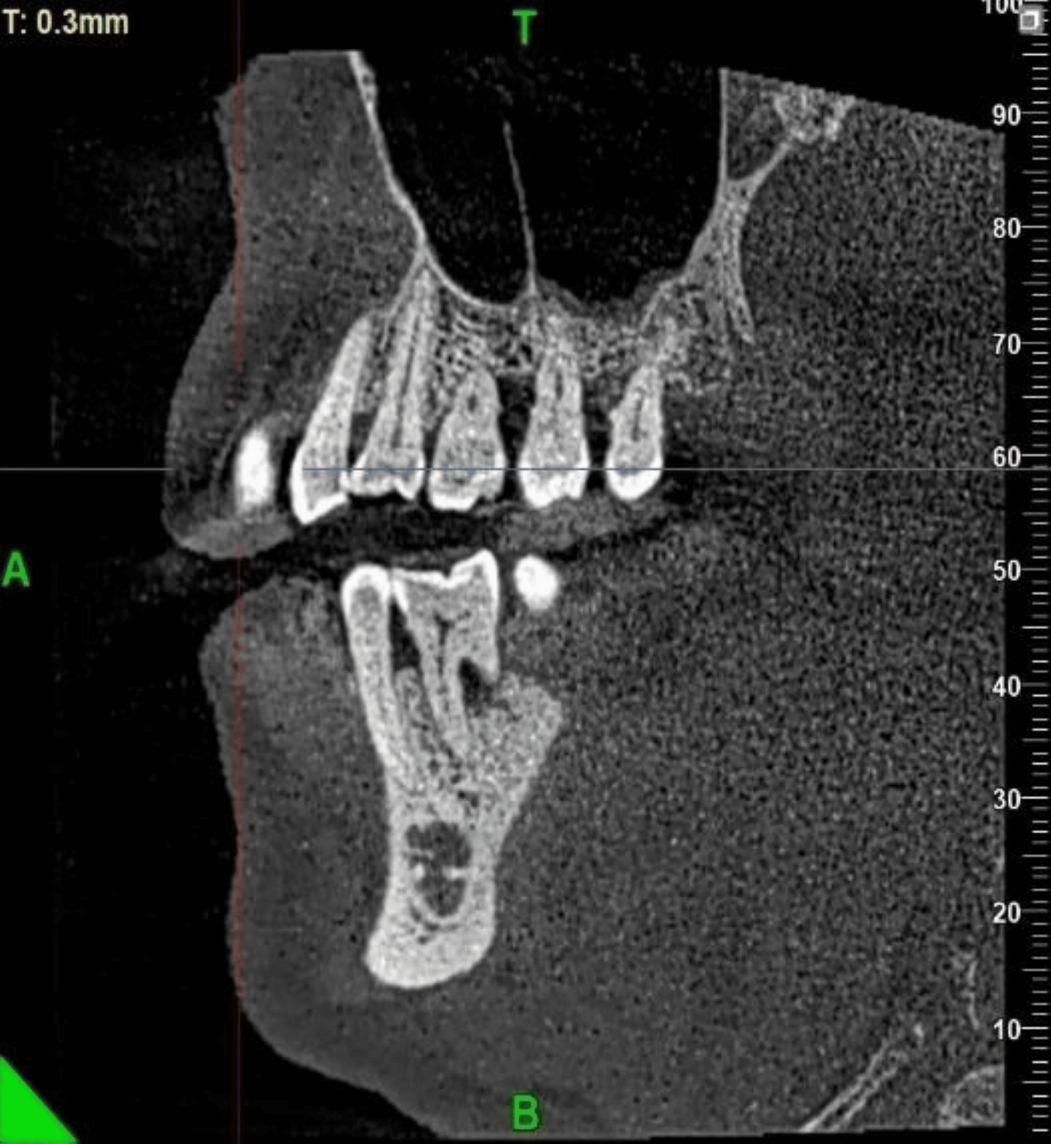
Sagittal view showing the location of septa in the middle

Orientation of septa

The orientations of the septa were recorded as either bucco-palatal (from the buccal or palatal side of the sinus) or anteroposterior (from the anterior or posterior wall of the sinus) in the axial view.

Classification and perforation risk

The types of septa and risk of Schneiderian membrane perforation were classified and evaluated according to a modification of Al-Faraje [8] introduced by Khalighi Sigaroudi et al. [9].

Statistical analysis

The study utilized Microsoft Excel 2007 for data entry and IBM SPSS Statistics for Windows, Version 23.0 (Released 2015; IBM Corp., Armonk, New York, United States) for analysis, employing descriptive statistics such as frequency and percentage to summarize the data. The chi-squared test was applied to examine categorical variables, with a significance level set at 5%. This statistical method was used to compare observed data with expected data based on a hypothesis, evaluate the fit of a statistical model to observed data, and test the association between variables in two-way tables. By calculating the chi-squared test statistic, the study determined the significance of the results, with a p-value of less than 0.05 indicating statistical significance.

## Results

In the study, the mean age was found to be 48.92±1.43 years in males and 49.59±1.54 years in females where the majority of images were of males at 56.33% followed by females which were 43.67%, respectively (Table 1). On age distribution analysis, it was observed that predominant images, i.e., 48%, of the total images fall within the age range of 21-50 years followed by the 60-70-year age group, with 21% of the total images. On image analysis, it was found that the majority (52.7%) were dentate, followed by partially edentulous (43%), whereas the smallest percentage (4.3%) were completely edentulous.

**Table 1:** Demographic details of study participants

Age (above 18 years)	Males	Females	Total
Number	169	131	300
Mean age	48.92±1.43	49.59±1.54	

Septa were present in 21.33% of the population, whereas in 78.67%, they were absent. On examination of different configurations, it was found that 90.63% of the subjects had a single septum followed by 7.81% which had two septa, and at least 1.56% had three septa in one sinus, which shows that there is a significant difference in configurations of septa (Table 2).

**Table 2:** Prevalence of septa with different configurations among the study population

	Configuration	N	Percentage
Prevalence of septa among the study population	Septa present	64	21.33%
	Septa absent	236	78.67%
Different septa configurations	One septum in one sinus	58	90.63%
	Two septa in one sinus	5	7.81%
	Three septa in one sinus	1	1.56%

As regards the orientation of septa, 93.75% exhibited a buccolingual orientation, while 6.25% displayed an anteroposterior orientation (Table 3). This shows that bucco-palatal orientation is more prevalent than anteroposterior orientation and shows a highly significant difference. About 20.1% of septa were in the left sinus and 22.5% in the right sinus, which did not show any significant difference. Among the study sample, the maximum height of the septa observed is 19.50 mm, whereas the minimum height observed is 1.90 mm with a mean height of 6.16 mm. When the age prevalence of septa was analyzed, it was found that maximum prevalence was seen in the 21-30-year age group, at 26%, while the lowest prevalence of septa was recorded at 15.6% in the older age group, i.e., 51-60-year-olds. However, on the basis of sex, it was found that septa were predominant in females (23.7%), whereas only 19.5% of males in the study population had septa.

**Table 3:** Orientation of septa

Orientation	N	Percentage	P-value
Anteroposterior	4	6.25%	0.001
Bucco-palatal	60	93.75%	

Table 4 shows the location of septa: 48.44% were situated in the middle, 32.81% were posteriorly located, and only 9.37% were anteriorly positioned, whereas 9.37% of the cases exhibited multiple septa in various locations, with highly significant differences among the locations of septa.

**Table 4:** Location of septa

Location	N	Percentage	P-value
Anterior	6	9.37%	0.001
Middle	31	48.44%	
Posterior	21	32.81%	
Multiple	6	9.37%	

The distribution of dentition revealed that the middle region showed the highest prevalence for both dentulous and partially dentulous conditions, whereas edentulous status was predominantly observed in the posterior region (Table 5).

**Table 5:** Prevalence of septa based on the side of the sinus and dentition *p<0.05 indicating statistical significance using the chi-squared test

		Septa present		Chi-squared value	P-value
		N	Percentage		
Prevalence based on the side of the sinus	Left	30	20.1%	0.25	0.615
	Right	34	22.5%		
Prevalence based on dentition (maxillary posterior region)	Edentulous	1	7.7%	0.77	0.234
	Partially dentulous	28	21.7%		
	Dentate	35	22.2%		

Table 6 shows the risk of perforation, revealing that Class III has the highest perforation with 48.4%, followed by Class I with 37.5%. Classes II and IV had relatively lower perforations at 3.1% and 4.6%, respectively, while Classes V and VI had no individual representation. A significant difference was observed among the seven classes based on the risk of perforation. Regarding the level of risk of perforation, the moderate level showed the highest perforation risk at 48.4%, followed closely by the low level at 46.8% and the high level which showed the lowest perforation risk at 4.6% with no significant difference among them. Gender distribution of the risk of perforation revealed that in the low risk of perforation, females were higher than males, at 53.4% and 46.6%, respectively. However, in the moderate risk and high risk of perforation groups, males constituted the majority. This reveals no significant difference in the sex distribution of the risk of perforation.

**Table 6:** Risk and level of risk of perforation *p<0.05 indicating statistical significance using the chi-squared test

		N	Percentage	Chi-squared value	P-value
Risk of perforation	Class I	24	37.5%	107.28	0.001
	Class II	2	3.1%		
	Class III	31	48.4%		
	Class IV	3	4.6%		
	Class V	0	0%		
	Class VI	4	6.2%		
	Class VII	0	0%		
Level of risk of perforation	Low	30	46.8%	23.65	0.000
	Moderate	31	48.4%		
	High	3	4.6%		

Table 7 presents the risk of perforation based on dental status, which revealed that at the low level, the majority were dentate (63.3%), followed by partially dentulous (36.7%), with no representation of edentulous. At the moderate level, both dentate and partially dentulous categories were evenly distributed at 48.3%, with a small representation of edentulous at 3.2%. The high level showed a predominance of partially dentulous at 66.7%, followed by dentate at 33.3%, with no representation of edentulous.

**Table 7:** Risk of perforation based on dental status *p<0.05 indicating statistical significance using the chi-square test

	Dentate		Partially dentulous		Edentulous		Total		Chi-squared value	P-value
	N	Percentage	N	Percentage	N	Percentage	N	Percentage		
Low	19	63.3%	11	36.7%	0	0%	30	100%	2.80	0.590
Moderate	15	48.3%	15	48.3%	1	3.2%	31	100%		
High	1	33.3%	2	66.7%	0	0%	3	100%		

Table 8 presents the risk of perforation based on location, which reveals that at the low level, the middle category has the highest perforation risk (54.8%), followed by posterior (32.2%), with anterior having the lowest risk (12.9%). At the moderate level, the middle category remained predominant (61.2%), followed by posterior (35.4%), with anterior having the lowest risk perforation (6.4%). At the high level, there's an equal distribution across all categories, with each representing 20% of the total.

**Table 8:** Risk of perforation based on location *p<0.05 indicating statistical significance using the chi-squared test

	Anterior		Middle		Posterior		Total		Chi-squared value	P-value
	N	Percentage	N	Percentage	N	Percentage	N	Percentage		
Low	4	12.9%	17	54.8%	10	32.2%	31	100%	1.24	0.824
Moderate	2	6.4%	19	61.2%	11	35.4%	31	100%		
High	1	20%	3	60%	1	20%	5	100%		

Table 9 presents the risk of perforation based on orientation, which reveals that in the low and moderate levels, the majority exhibited bucco-palatal orientation, representing 90.3% and 96.7%, respectively, while a smaller proportion demonstrated anteroposterior, representing 9.7% and 3.3%, respectively. At the high level, all individuals exhibited bucco-palatal, constituting 100% of the total, with no risk of perforation of anteroposterior. The study sample showed that the mean age at risk of perforation was 48.35 years.

**Table 9:** Risk of perforation based on orientation *p<0.05 indicating statistical significance using the chi-squared test

	Bucco-palatal		Anteroposterior		Total		Chi-squared value	P-value
	N	Percentage	N	Percentage	N	Percentage		
Low	28	90.3%	3	9.7%	31	100%	1.32	0.515
Moderate	30	96.7%	1	3.3%	31	100%		
High	3	100%	0	0%	0	100%		

## Discussion

Apart from oro-antral fistula and sinus inflammation, perforation of the Schneiderian membrane is the most common complication (7-35%) during surgery in the maxillary region. Septa, which are bony structures dividing the sinus cavity, can lead to perforation of the Schneiderian membrane, and high septa can pose challenges during sinus lift or implant surgery by hindering the placement of implants or complicating the elevation of the sinus membrane for grafting. The presence of septa in maxillary sinus surgeries warrants careful consideration [3].

Panoramic imaging is frequently employed as a radiographic method in dentistry [10]. However, septa may not always be visible in all images because of the limitations of two-dimensional radiography [11]. Certain types of septa may not be apparent in the frontal plane, necessitating examination of sagittal and axial planes to detect them effectively [12].

CBCT possesses the requisite resolution to effectively identify and visualize osseous intricacies within the maxillary sinus. Moreover, it offers biological advantages over conventional computed tomography [13].

This study has three primary objectives. First, we aimed to determine the prevalence, height, location, and orientation of septa within the maxillary sinus. Second, we sought to assess the risk of Schneiderian membrane perforation using the classification system proposed by Khalighi Sigaroudi et al. [9], which provides a structured approach for predicting perforation risk during surgical procedures. Finally, we investigated the potential correlations between the risk of perforation and patient demographics, including age, sex, and dental status. Through these objectives, this study aimed to advance our understanding of septa-related complications in maxillary sinus surgeries, facilitating informed risk assessment and the development of tailored surgical approaches. In addition, elucidating the relationship between septa morphology and perforation risk can guide the formulation of customized surgical protocols, thereby minimizing intraoperative complications. Exploring the impact of patient-specific factors, such as anatomical variability and systemic health conditions, on septa-related risks will contribute to personalized treatment planning and improved surgical outcomes [14]. By integrating multidisciplinary approaches and evidence-based strategies, this study strives to establish a comprehensive framework for risk assessment and management in maxillary sinus surgeries, ultimately enhancing patient safety and satisfaction. In the present study, a total of 300 maxillary sinuses from CBCT images were analyzed, of which 169 scans (56%) were of males with a mean age of 48 years and 131 (44%) were of females with a mean age of 49 years.

This study revealed that the prevalence of sinus septa was 21.33% in the sinus segments, which is consistent with the findings of Alhumaidan et al. [15] and Mirdad et al. [16] but in contrast with Assari et al. [6] and Altayar et al. [17]. The differences in the prevalence of sinus septa between the studies could be attributed to variations in the study population, sample size, methodology, observer bias, and geographical variation.

In this study, in 90.63% of the subjects, a single septum was observed in one sinus, whereas in 7.81% of the subjects, two septa were present in one sinus. Additionally, one subject exhibited three septa in one sinus, which is consistent with the findings of Souza et al. [18] and Dandekeri et al. [19] but in contrast with Tadinada et al. [20].

In the present study, across all age groups, septal prevalence showed no significant difference, with the lowest occurrence (0%) in the 10-20-year age group, while the highest (26%) was observed in the 21-30-year age group. Similar results were observed by Dandekeri et al. [19] and Tadinada et al. [20] but in contrast to the findings of Şimşek Kaya et al. [21].

In this study, the prevalence of septa did not significantly differ between males (19.5%) and females (23.7%). This was similar to the findings of Tadinada et al. [20] but in contrast to the findings of Sakhdari et al. [22].

In this study, septal presence did not exhibit a significant discrepancy between the left sinuses (20.1%) and the right sinuses (22.5%). This was similar to the findings of Tadinada et al. [22]. However, this is in contrast with Demirkol and Demirkol [23] which showed 52.8% were located on the right side and 47.2% were located on the left side. The difference in septal prevalence may stem from variations in the sample size, methodology, interpretation, and anatomical diversity, among other factors.

In this study, the highest number of septa was observed in dentate patients, followed by partially edentulous and edentulous patients. This is consistent with the findings of Orhan et al. [24] and Toraman Alkurt et al. [25].

In the present study, the observed septa heights range from 1.90 mm to 19.50 mm, with a mean height of 6.16 mm. This is in accordance with Jang et al. [26] and Al-Zahrani et al. [2] but in contrast with Wang et al. [27] and Taleghani et al. [28].

The data indicated a higher prevalence of septa in the bucco-palatal orientation (93.75%) compared to the anteroposterior orientation (6.25%). Similar findings were reported by Irinakis et al. [29] and Alhumaidan et al. [15], but opposite findings were reported by Talo Yildirim et al. [30].

Regarding the location of septa, 48.44% were situated in the middle, 32.81% were posteriorly located, and 9.37% were anteriorly positioned. Additionally, 9.37% of the cases exhibited multiple septa present in various locations. This was similar to the findings of Orhan et al. [24] and Demirkol and Demirkol [23] but in contrast to Jang et al. [26].

In our study, maximum septa were seen in Class III based on the classification of risk of perforation, whereas the least prevalence was seen in Classes V and VII, which is similar to the results of Alhumaidan et al. [15], who also reported a maximum prevalence of Class III but was not consistent with the findings of Khalighi Sigaroudi et al. [9], who reported a maximum prevalence of Class I. The maximum number of septa falls into the category of moderate risk of perforation, which is in harmony with Alhumaidan et al. [15] but opposite to Khalighi Sigaroudi et al. [9]. The least prevalence was seen in the high risk of perforation in our study and also in Alhumaidan et al. [15] and Khalighi Sigaroudi et al. [9]. On the basis of sex, a greater number of males fell into the category of moderate risk, whereas more females were observed in the category of low risk. However, in a study by Alhumaidan et al. [15], the maximum number of both males and females had a moderate risk, whereas according to Khalighi Sigaroudi et al. [9], the maximum number of both males and females was low. In our study, a higher risk of perforation was observed in the dentate patients. Based on location, the maximum number of septa that were placed anteriorly had low risk, whereas more septa in the middle and posterior regions had low risk. Based on orientation, the maximum number of septa of bucco-palatal orientation had moderate risk, whereas the maximum number of septa of anteroposterior orientation had low risk. This is similar to Alhumaidan et al. [15]. Variability in sample characteristics, methodology, and interpretation criteria can lead to similarities and differences in the findings among different studies.

Furthermore, understanding the relationship between the characteristics of maxillary sinus septa and the risk of Schneiderian membrane perforation is crucial for effective preoperative planning and reduction of surgical complications. Analyzing the correlations between patient demographics and septa-related risks could also enable the development of personalized treatment approaches, thereby optimizing clinical outcomes. Utilizing advanced imaging techniques, such as CBCT, allows for improved diagnostic accuracy and refinement of surgical methodologies. By incorporating a thorough analysis of septa-related variables, this study seeks to enhance clinical practices, mitigate risks, and ultimately improve patient safety and outcomes in maxillary sinus surgeries [7].

Limitations of the study

This study analyzed 300 maxillary sinuses using CBCT scans to estimate the prevalence, height, location, orientation, and risk of perforation of maxillary sinus septa. However, it had limitations, including its retrospective design, single-center data, limited sample size, and lack of clinical correlation and longitudinal follow-up. These limitations highlight areas for future research to improve the understanding of maxillary sinus septa and their clinical significance.

## Conclusions

This study demonstrated a notable prevalence of maxillary sinus septa, observed in 21.33% of the cases, with single septa being the most common. These findings highlight that septa are frequently situated in the middle region of the sinus and predominantly exhibit a bucco-palatal orientation. The risk of perforation of the Schneiderian membrane varies with septa configuration, orientation, and height, with Class III septa showing the highest risk. CBCT is an invaluable tool for identifying these anatomical features, aiding precise preoperative planning and risk assessment. By providing detailed insights into the prevalence, configuration, and associated risks of maxillary sinus septa, this study contributes to enhancing surgical strategies, minimizing complications, and improving patient outcomes in maxillary sinus surgery.
